# Effects of Sheep Sires on Muscle Fiber Characteristics, Fatty Acid Composition and Volatile Flavor Compounds in F_1_ Crossbred Lambs

**DOI:** 10.3390/foods11244076

**Published:** 2022-12-16

**Authors:** Zengkui Lu, Yaojing Yue, Haina Shi, Jinxia Zhang, Tiaoguo Liu, Jianbin Liu, Bohui Yang

**Affiliations:** 1 Key Laboratory of Animal Genetics and Breeding on the Tibetan Plateau, Ministry of Agriculture and Rural Affairs, Lanzhou Institute of Husbandry and Pharmaceutical Sciences, Chinese Academy of Agricultural Sciences, Lanzhou 730050, China; 2 Sheep Breeding Engineering Technology Research Center of Chinese Academy of Agricultural Sciences, Lanzhou 730050, China; 3 Institute of Agricultural Sciences of Qingyang, Qingyang 745000, China

**Keywords:** crossbred lamb, hybridization, muscle fiber characteristic, fatty acid composition, volatile flavor compound

## Abstract

Crossbreeding significantly improves meat production performance in sheep; however, whether hybridization changes the meat quality characteristics of lambs is uncertain. We analyzed the effects of three different hybrid sires on muscle fiber characteristics (MFCs), fatty acid composition (FAC), and volatile flavor compounds (VFCs) in lambs under identical feeding conditions. Compared with those of purebred lambs, the muscle fiber diameter and cross-sectional areas of the crossbred lambs were significantly decreased (*p* < 0.05), and the collagen fiber content was significantly increased (*p* < 0.05). The numbers and area ratios of the fast and slow muscle fibers did not significantly differ between the purebred and crossbred lambs, but the expressions of four *MyHC* gene types differed significantly (*p* < 0.05). Twenty-three fatty acids were identified in both the purebred and crossbred lambs, of which thirteen were differentially expressed (*p* < 0.05). Saturated fatty acid (SFA) contents in the crossbred lambs were significantly increased (*p* < 0.05), whereas the monounsaturated fatty acid content was significantly decreased (*p* < 0.05). Polyunsaturated fatty acid/SFA and n-6/n-3 ratios were significantly lower in the crossbred lambs than in the purebred lambs (*p* < 0.05). Twenty-five VFCs were identified among the three hybrids, and aldehydes were the main VFCs. Eleven VFCs were differentially expressed in the crossbred lambs (*p* < 0.05). Hybrid sires affected the MFCs, FAC, and VFCs of the F_1_ lambs, thus providing a reference for high-quality mutton production.

## 1. Introduction

As people’s quality of life improves, they have greater food requirements and pay more attention to high-quality protein intake. Mutton is popular for its high protein and low cholesterol content [1,2]. Although China is a large producer and consumer of mutton, the overall mutton industry lags far behind that of developed countries with regard to animal husbandry. Marketed mutton is mainly local mutton; the carcasses can be large, small, fat, or thin, and the output of high-quality mutton cannot meet the growing rigid demand of the market. This has resulted in the growing trend toward crossbreeding to produce fatty lambs in the Chinese mutton industry.

Hybridization produces heterosis and is a main biological method for improving production performance. Compared with their parents, hybrid progenies exhibit rapid growth and high stress resistance [3]. In animal husbandry in developed countries, over 80% of commercial pigs are hybrids, and heterosis is widely used in beef cattle and mutton sheep. Hybridization improves the production efficiency of livestock and poultry, but whether the increased production performance affects meat quality is uncertain. Meat quality is a comprehensive characteristic and includes appearance, physical indexes, nutritional composition, fatty acid composition (FAC), and volatile flavor compounds (VFCs) [4,5]. Muscle fibers are the basic units of muscle tissue, and the histological characteristics of muscle fibers are closely related to meat quality, reflect the internal structure of the meat, and partly reflect the connective tissue, myofibril, and fat content in the muscle [6,7,8]. Studies have shown that hybridization significantly changes the FAC and VFCs of livestock and poultry muscle and can be used to increase or decrease specific meat quality characteristics [9,10,11]. However, little is known regarding whether crossbreeding sheep changes the muscle fiber characteristics (MFCs), FAC, and VFCs.

In the United Kingdom, Southdown is considered the best breed for meat quality owing to its ideal meat body structure. Southdown sires are usually used as the terminal cross in mutton sheep crosses. Suffolk sheep offer fast production development and good meat production performance and are currently recognized worldwide as a terminal crossbreed for improved meat sires. Hu sheep exhibit perennial estrus, high fecundity, high lactation, good parenting traits, and strong adaptability; therefore, they are often used as economic crossbreeding ewes for mutton sheep. In this study, three sheep breeds of Hu sheep, Southdown, and Suffolk were selected as sires under the same feeding conditions and crossed with Hu sheep ewes to compare and analyze the differences in MFCs, FAC, and VFCs of 12-month-old hybrid lambs. The results will provide a good reference for effective use of heterosis to improve local breeds.

## 2. Materials and Methods

### 2.1. Animals and Sample Collection

The lambs chosen in this experiment were approved by the Animal Administration and Ethics Committee of Lanzhou Institute of Husbandry and Pharmaceutical Science of Chinese Academy of Agricultural Science (Permit No. SYXK-2019-010). Sample size was determined as the minimal number of animals that would provide statistical power to detect a group difference. Three replicates were performed in each group, which meet the principles of the experimental design of laboratory experiments. During the experiment, lambs may be removed for ethical reasons, so three more lambs were added to each group to guarantee the number of lambs in each group reach a number of six lambs. Six F_1_ lambs (ram) were selected from each group of Hu × Hu (HH), Southdown × Hu (NH), and Suffolk × Hu (SH) sheep, and each lamb came from a different sire. All lambs were selected from one of the twins. All lambs had the same birth date, feeding level, and feeding management procedures. At 12 months of age, three lambs per group with consistent growth were randomly selected for slaughtering. The longissimus dorsi muscle was collected and divided into two parts: one part was stored in 4% paraformaldehyde solution and the other part was stored in a −80 °C freezer.

### 2.2. Histological Analysis of the Muscle

For hematoxylin and eosin staining, the longissimus dorsi tissue was stored in 4% paraformaldehyde, and then dehydrated in gradient ethanol (75% 2 h→80% 45 min→95% 30 min→100% 30 min→100% 20 min), transparentized in gradient xylene (50% 30 min→100% 10 min), embedded in paraffin, and sectioned at ~5 µm thickness with at least three sections per sample. The dewaxed sections were stained with hematoxylin (Sigma, Shanghai, China) for 2 min, differentiated for 2 s in differentiation solution, and stained with eosin (Sigma) for 2 min. The sections were dehydrated and transparentized, sealed with resin, and preserved. A microscope (Olympus Dp71, Tokyo, Japan) was used to view the sections, and CaseViewer software (3DHISTECH, Budapest, Hungary) was used to scan the images. Four visual fields were selected per section, and the diameter, cross-sectional area, and fiber density per unit area were measured and analyzed using ImageJ software. In ImageJ, a double loop over the corner pixels algorithm is used for calculation. In short, (1) Open the target image, File→Open; (2) Set the scale, Analyze→Set Scale; (3) Gray image, Image→Type→8-bit; (4) Set threshold value, Image→Adjust→Threshold, default parameter; (5) Process→Binary→Watershed; (6) Analyze→SetMeasurement, check Fee’s diameter; (7) Analyze→Analyze Particles, check Display results, Clear results, Summarize, and Exclude on edges. In the output results, Feret is the maximum Feret diameter, FeretX and FeretY are the top-left endpoint coordinates of the maximum Feret diameter, FeretAngle is the angle between the maximum Feret diameter and X positive semi-axis, and minFeret is the smallest Feret diameter. The geometric mean of the maximum and minimum Ferrett diameters is used to represent the diameter size. A minimum of 200 muscle fibers was used for each sample calculation.

For Masson staining, dewaxed sections were stained with Weigert’s iron hematoxylin (Sigma) for 5 min, differentiated in solution for 5 s, stained with acid fuchsin (Sigma) for 7 min, treated with phosphomolybdic acid (Sinopharm Chemical Reagent, Shanghai, China) solution for 4 min, stained with aniline blue (Sigma) for 5 min, and differentiated with 1% glacial acetic acid (Sinopharm Chemical Reagent) for 1 min. The sections were sealed with resin, a microscope was then used to view the sections, and CaseViewer software was used to scan the images. Four visual fields were selected per slice, and the collagen fiber content per unit area was calculated and analyzed using ImageJ software.

For fast and slow muscle immunofluorescence staining, the dewaxed sections were heated with EDTA antigen repair solution (Servicebio, Wuhai, China) in a microwave oven for 10 min, cooled, and washed with PBS (Sigma). The sections were immersed in 3% hydrogen peroxide (Servicebio) at 25 °C for 25 min. After washing again, the sections were incubated with bovine serum albumin (Sigma) for 30 min, and the excess liquid was removed, then added fast primary antibody (Myosin-1, Servicebio, GB112130, 1:3000) and incubated overnight in a wet box at 4 °C. Next, the sections were washed three times, and then incubated with HRP-labeled secondary antibody (Servicebio, GB23303, 1:500) for 50 min at room temperature. After being washed again with PBS, the sections were incubated with CY3-TSA (Servicebio) for 10 min at 25 °C in the dark. The sections were washed with TBST (Servicebio) and heated with EDTA antigen repair solution in a microwave oven for 10 min. After cooling, the excess liquid was removed again, and the sections were incubated with slow primary antibody (Myosin-7, Servicebio, GB111857, 1:500) overnight in a 4 °C wet box, then washed with PBS and incubated with HRP-labeled secondary antibody (Servicebio, GB25303, 1:400) for 50 min at room temperature. After the excess liquid was removed, the sections were incubated with DAPI solution (Servicebio) for 10 min at 25 °C in the dark, then sealed with antifluorescence quenchant and preserved. A microscope was used to view the sections, and CaseViewer software was used to scan the images. Four visual fields were selected per section, and the numbers and areas of fast and slow muscles per unit area were calculated and analyzed using ImageJ software.

### 2.3. Fluorescence Quantitative PCR

Total RNA was extracted with TRlzol reagent (TransGen Biotech, Beijing, China) and reverse transcribed into cDNA using a cDNA synthesis kit (TransGen Biotech). Primers were designed and synthesized using Primer 5 software (Table S1). TransStart Green qPCR SuperMix (TransGen Biotech) was used to perform qRT-PCR in a LightCycler 480II instrument (Roche, Basel, Switzerland), and each sample was run four times. *ACTB* was used as an internal reference gene, and the relative expression of the target gene was calculated via the 2^−ΔΔCt^ method.

### 2.4. Fatty Acid Composition Analysis

Three grams of muscle sample was weighed and ground into powder with liquid nitrogen. Fat extract (10 mL, chloroform:methanol = 2:1, Sinopharm Chemical Reagent) was added during grinding. After centrifugation at 4000 r/min for 10 min, the upper liquid was discarded, and 2 mL chloroform (Sinopharm Chemical Reagent) and 4 mL distilled water were added. Next, 1 mol/L sodium hydroxide methanol (Sinopharm Chemical Reagent) solution was added, and the sample was incubated in a water bath at 60 °C for 1 h. After cooling to room temperature, 2 mL 10% sodium bisulfate (Sinopharm Chemical Reagent), 2 mL 25% sodium chloride (Sinopharm Chemical Reagent), 6 mL distilled water, and 3 mL n-hexane (Sigma) were added and incubated for 10 min. The upper liquid was added to a test tube containing anhydrous sodium sulfate and filtered through a 0.22 µm organic membrane (Sigma) for gas chromatographic (GC) analysis (Agilent 7890A, California, CA, USA).

The GC conditions were an SP-2560 chromatographic column (100 m × 0.25 mm × 0.2 µm), injection temperature: 260 °C, nitrogen flow rate: 1 mL/min, injection volume: 1.0 µL, and split ratio: 20:1. The heating program was an initial temperature of 140 °C for 5 min, which was increased at 2 °C/min to 200 °C for 5 min, and then at 6 °C/min to 230 °C for 20 min. The fatty acids were qualitatively determined according to the retention times of 38 known fatty acid methyl esters and quantified according to the peak area.

### 2.5. Identification of Volatile Flavor Compounds

GC-IMS (gas chromatography-ion mobility spectroscopy; G.A.S. FlavourSpec^®^, Dortmund, Germany) equipped with SE-54 capillary columns were adopted to determine the VFCs in the mutton. A muscle sample of three grams was placed in a 20 mL headspace vial and incubated at 80 °C for 15 min. At 85 °C, the injection needle was used to inject 500 µL in non-shunt mode. The conditions were set as follows: 60 °C of column temperature; 45 °C of drift tube temperature; a drift gas flow rate of 150 mL/min; and the nitrogen carrier gas purity was 99.999%. The GC column flow rate was 2 mL/min for 2 min, then kept 20 mL/min for 8 min, and finally 100 mL/min for 15 min. N-keto C4–C9 was used as the external standard for calculation of retention index. The VFCs were identified by comparison of the drift time standards in the RI (retention index) and GC-IMS libraries.

The NIST and IMS databases were built in GC-IMS Library search software and used to analyze flavor substances. The fingerprints of the VFCs were constructed using Reporter and Gallery plug-ins in LAV (laboratory analytical viewer) software, and PCA (principal component analysis) was conducted using Dynamic PCA plug-ins.

### 2.6. Statistical Analysis

Three lambs were randomly selected from each group, and three replicates were performed per lamb. We first calculated the average value of each of the three replicates per lamb, and then calculated the overall average value. All data in the tables and figures were represented by mean ± SD and were statistically analyzed by one-way analysis of variance in SPSS17.0 software at *p* < 0.05 as the level of significance.

## 3. Results

### 3.1. Muscle Fiber Characteristics of the Three Lamb Hybrids

**Figure 1:**
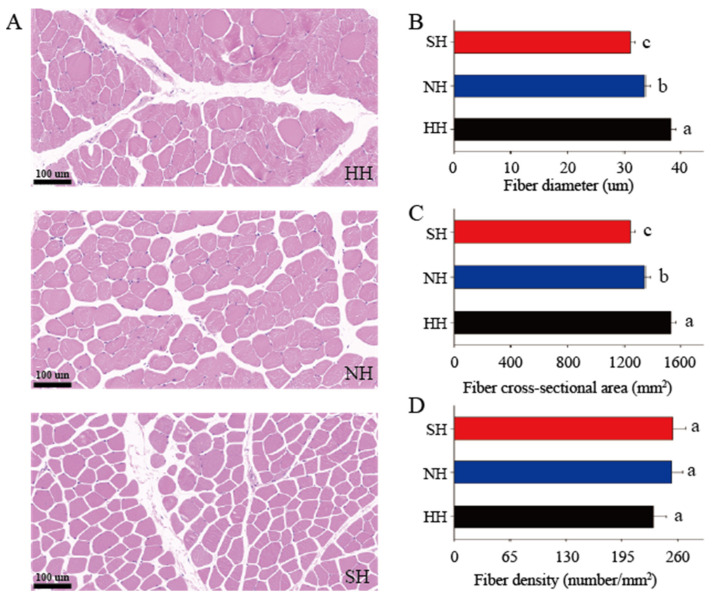
Muscle fiber morphology traits in F_1_ crossbred lambs. (**A**) Hematoxylin and eosin staining. (**B**) Muscle fiber diameter. (**C**) Muscle fiber cross-sectional area. (**D**) Muscle fiber density. At least 200 muscle fibers were analyzed in each sample. Scale bars, 100 μm. All data were expressed as mean ± SD (*n* = 3). Different letters indicate significant differences (*p* < 0.05). HH: Hu × Hu, NH: Southdown × Hu, SH: Suffolk × Hu.

The muscle fibers of the lamb hybrids are displayed in Figure 1A. Under light microscopy, the nuclei stained blue, but the muscle fibers stained red. The diameter and cross-sectional areas of the muscle fibers were significantly larger in the HH group than in the NH and SH groups (*p* < 0.05), and those in the NH group were significantly larger than those in the SH group (*p* < 0.05, Figure 1B,C). Muscle fiber densities did not significantly differ among the groups (Figure 1D).

**Figure 2:**
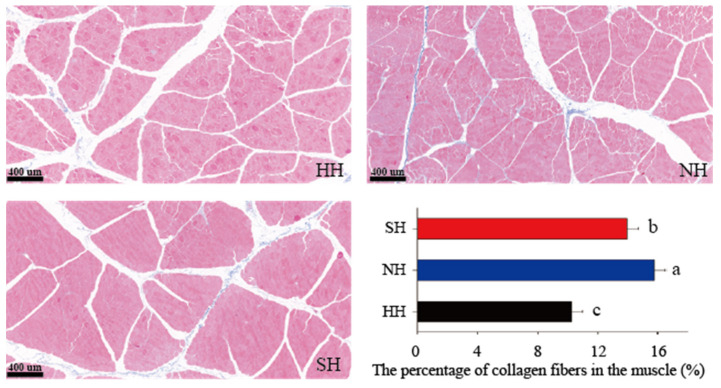
Collagen fiber content in F_1_ crossbred lambs using masson staining. Scale bars, 400 μm. All data were expressed as mean ± SD (*n* = 3). Different letters indicate significant differences at *p* < 0.05. HH: Hu × Hu, NH: Southdown × Hu, SH: Suffolk × Hu.

Masson staining was used to detect the collagen fiber content in the muscle fibers of the hybrid lambs. Under light microscopy, collagen fibers stained blue, but muscle fibers stained red (Figure 2 and Figure S1). The collagen fiber contents were 10.23%, 15.78%, and 13.96% in the HH, NH, and SH groups, respectively (Figure 2). The collagen fiber content was significantly larger in the NH group than in the HH and SH groups (*p* < 0.05), and that in the SH group was significantly larger than that in the HH group (*p* < 0.05).

**Figure 3:**
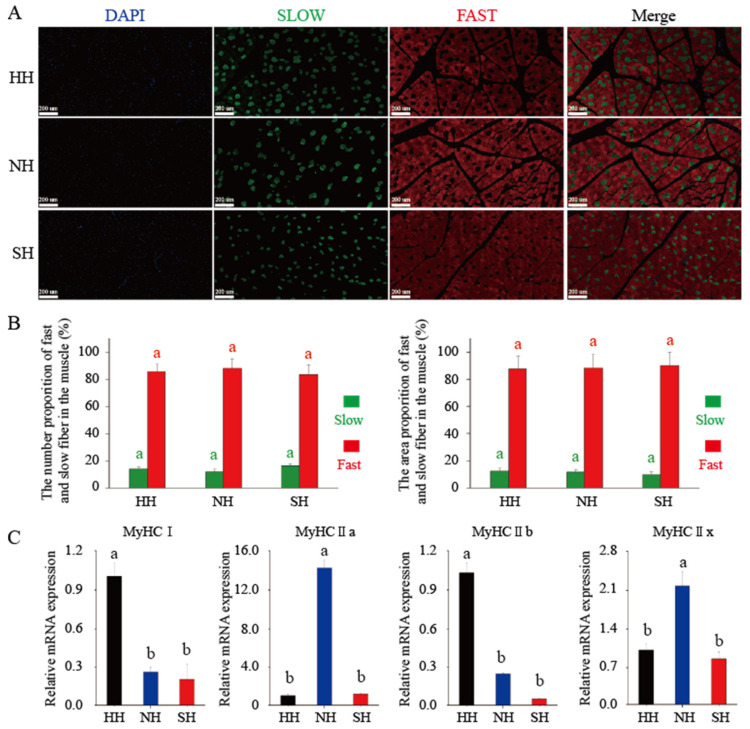
Muscle fiber type composition in F_1_ crossbred lambs. (**A**) Immunofluorescence staining detected the distributions of fast and slow muscle fibers. (**B**) The number and area proportion of fast and slow fiber in the muscle. (**C**) Expression of *MyHCs* genes in F_1_ crossbred lambs. Scale bars, 100 μm. All data were expressed as mean ± SD (*n* = 3). Different letters indicate significant differences at *p* < 0.05. HH: Hu × Hu, NH: Southdown × Hu, SH: Suffolk × Hu.

Immunofluorescence staining was selected to detect the distributions of fast and slow muscle fibers in the lamb muscle fibers. Under fluorescence microscopy, slow muscle fibers stained green, and fast muscle fibers stained red (Figure 3A). The numbers and area ratios of the slow and fast muscle fibers did not significantly differ among the three groups (Figure 3B). *MyHC* molecular typing was used to quantitatively classify the muscle fiber types (Figure 3C). The expression of *MyHCI* was significantly higher in the HH group than in the groups of NH and SH (*p* < 0.05). *MyHCIIa* gene expression was significantly higher in the NH group than in the HH and SH groups (*p* < 0.05). The expression of *MyHCIIb* was significantly higher in the HH group than in the NH and SH groups (*p* < 0.05), and *MyHCIIx* gene expression was dramatically higher in the NH group than in the HH and SH groups (*p* < 0.05).

### 3.2. Fatty Acid Composition Analysis and Contents in the F_1_ Crossbred Lambs

Twenty-three fatty acids were detected in the three mutton hybrids (Table 1): eight kinds of monounsaturated fatty acids (MUFAs), ten kinds of saturated fatty acids (SFAs), and five kinds of polyunsaturated fatty acids (PUFAs). SFAs are mainly composed of C18 and C15, MUFAs are dominated by C18:1n9t and C22:1n9, and PUFAs are dominated by C20:4n6. For SFAs, C10:0, C12:0, and C20:0 levels were definitely higher in the HH group than in the groups of NH and SH (*p* < 0.05), and C18:0 levels were significantly higher in the SH and NH groups than in the HH group (*p* < 0.05). For MUFAs, C14:1, C18:1n9c, C18:1n9t, C20:1, and C22:1n9 were significantly higher in the HH group than in the NH and SH groups (*p* < 0.05). For PUFAs, C18:2n6t and C20:2 were significantly higher in the HH group than in the NH and SH groups (*p* < 0.05), and C18:2n6c and C18:3n3 were significantly higher in the SH and NH groups than in the HH group (*p* < 0.05). PUFA/SFA and n-6/n-3 ratios were significantly higher in the HH group than in the SH and NH groups (*p* < 0.05).

### 3.3. GC-IMS Topographic Maps of the F_1_ Crossbred Lambs

**Figure 4:**
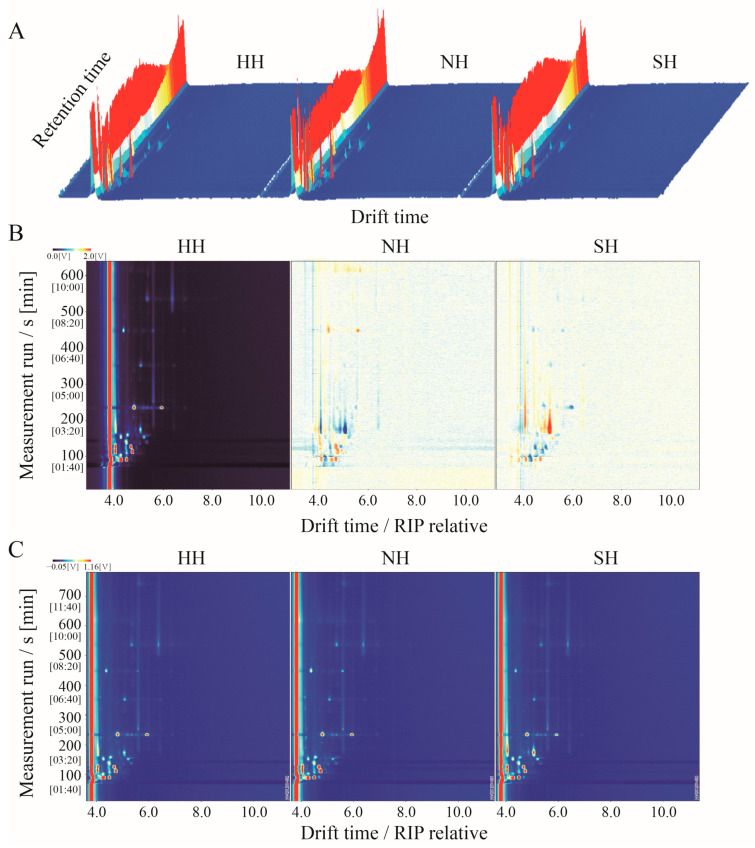
GC-IMS topographic maps in F_1_ crossbred lambs. (**A**) GC-IMS 3D spectrum. The x axis—the ion migration time; y axis—the GC retention time; z axis—the peak height of quantification. (**B**) GC-IMS 2D map. HH was regarded as the reference plot. The point colors represent the substance concentration: white—identical concentrations; blue—low concentrations; red—high concentrations. The vertical red line—the reactive ion peak (RIP) normalized to the drift time. Each point represents a volatile compound. (**C**) Ion migration spectra. HH: Hu × Hu, NH: Southdown × Hu, SH: Suffolk × Hu.

GC-IMS was used to detect VFCs in the three mutton hybrids. VFC content is expressed by a three-dimensional spectrogram, reflecting the group differences. The VFCs detected were similar among the three mutton types, but the signal intensity differed slightly (Figure 4A). To better compare the differences between groups, the HH group was selected as the reference group, and the two-dimensional topographic maps of the NH and SH groups were deduced (Figure 4B). For VFCs with the same composition, the background after deduction is white. When the substance content is higher than the reference value, it shows red; blue indicates that it is lower. Most signals are detected within 100–600 s of hold time and 1.0–1.5 s of drift time. Among the groups, some VFC signals disappeared or decreased, whereas others were enhanced, showing some differences. The ion migration map was drawn according to the retention time and drift time on three-dimensional and two-dimensional maps (Figure 4C).

### 3.4. Identification of Volatile Flavor Compounds in the F_1_ Crossbred Lambs and Construction of Fingerprints

The ion migration map revealed 25 target compounds identified by the GC-IMS library in the 3 mutton types (Table 2): 3 alcohols, 12 aldehydes, 7 ketones, 2 esters, and 1 pyrazine. Eight compounds were identified as both monomers and dimers. Six monomers (1-Propanol, Benzaldehyde, Methional, Heptanal, 3-Methylbutyraldehyde, and 2-Butanone) and five dimers (Benzaldehyde, Hexanal, Pentanal, 3-Methylbutyraldehyde, and 2-Butanone) differed significantly among the three hybrids (*p* < 0.05).

**Figure 5:**
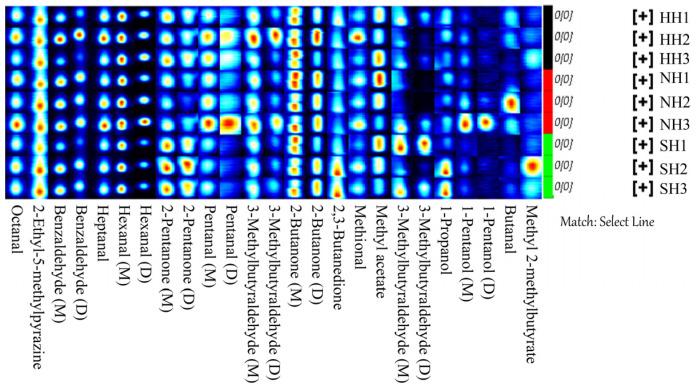
Fingerprint of volatile flavor compounds in F_1_ crossbred lambs.

**Figure 6:**
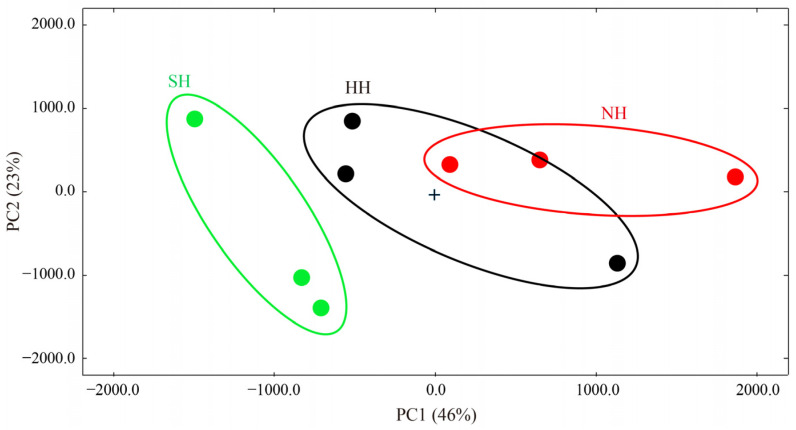
PCA of volatile flavor compounds in F_1_ crossbred lambs.

To better compare the compounds in the three hybrids, mutton fingerprints were constructed using LAV software. In Figure 5, each row represents a mutton hybrid, each column represents a compound, and signal differences are shown for each group. To better distinguish the mutton hybrid combinations, PCA was used to analyze the compounds. The contribution rate of PC1 was 46%, and that of PC2 was 23%, totaling 69% (Figure 6). In general, when the cumulative contribution rate ≥ 60%, the PCA model is considered the preferred separation model. Mutton VFCs differed significantly between the NH and SH groups, as shown by the PC1 value. On the PC1 axis, NH mutton was positive, and SH mutton was negative. However, the mutton VFCs were difficult to distinguish between the NH and SH groups using PC2. HH mutton was distinguishable from SH mutton. Therefore, the different sires significantly affected the VFCs in the mutton hybrids.

## 4. Discussion

China’s demand for high-quality mutton is increasing. Crossbreeding efficiently improves commercial carcasses in sheep production, and diversity between breeds and strains is obtained through heterosis. To our knowledge, studies on how crossbreeding affects the meat quality characteristics of sheep are lacking. Therefore, this study was performed to investigate the changes of different hybrid sheep sires on the MFCs, FAC, and VFCs of F_1_ offspring.

Muscle fiber accounts for 75–90% of muscle volume, and it plays a vital role in meat quantity and quality [12]. Studies have shown that many aspects of meat quality are related to the MFCs, specifically the total number of fibers, cross-sectional area, and fiber type [6,7,13]. In this study, the fiber diameter and cross-sectional area were significantly smaller in the NH and SH muscles than in the HH muscles, but there was no significant effect on fiber density. Generally, smaller muscle fiber diameters correspond to smaller cross-sectional areas and higher densities. Muscles with smaller muscle fiber diameters are reported to be juicier [14], possibly because muscle fiber size affects muscle growth potential and fiber bundle size, resulting in a significantly rougher cross section of meat [15,16]. Because MFCs are closely related to meat quality, many studies have confirmed that the meat quality of hybrid breeds is better than that of local breeds [17,18,19]. The number of muscle fibers is determined before birth, and later growth stages mainly exhibit hypertrophy and type transformation of muscle fibers. MFCs differed significantly among the hybrids, which may partly explain the differences in meat quality of different breeds. Therefore, breed is an important factor, and mutton quality can be changed by selecting different hybrid sires.

Collagen fibers are the main proteins in intramuscular connective tissue. Studies showed that the collagen fiber content was negatively correlated with meat tenderness, whereas the heat-soluble collagen fiber content was positively correlated with meat tenderness [20,21]. The proportion of total collagen fiber was significantly higher in the NH and SH groups than in the HH group; thus, the mutton tenderness in the NH and SH groups may have been worse. Additionally, collagen fiber content is reported to be positively correlated with the shear force of meat [22]; hence, the shear force of the mutton was smaller in the NH and SH groups than in the HH group. Shear force increases as the muscle fiber diameter increases, indicating that fiber diameter affects meat tenderness, and meat with a larger diameter is less tender [23]. This contradicts our result regarding estimation of tenderness using collagen fiber content, and further studies are needed that combine other meat quality indicators.

Muscle fibers are divided into four types according to their morphological and metabolic characteristics: slow oxidation type (I), rapid oxidation type (IIa), intermediate type (IIx), and rapid glycolysis type (IIb). Muscle fibers can be further divided into slow (I) and fast (IIa, IIx, and IIb) muscle fibers according to their contractile characteristics. In the current study, IIa and IIx antibodies in the sheep muscle fibers could not be easily discerned; therefore, the muscle fibers were divided into slow and fast muscle fibers only. Further distinguishing IIa, IIx, and IIb was impossible. Our results showed that the number and area of the slow muscle fibers accounted for ~10% of the total muscle fibers, which is similar to that in the longissimus dorsi of pigs [14]. Many studies have shown that the *MyHC* content is closely related to the compositions of muscle fiber types [12]. Therefore, we further used qRT-PCR to classify the muscle fiber types and found that the muscle fiber types changed with the different hybrid sires. Four muscle fiber types were detected in the three mutton hybrids, but previous studies found that *MyHCIIb* was not expressed in sheep or horse muscle fibers [24], which contradicts our results. However, *MyHCIIb* expression was detected in sheep muscle fibers in several recent studies [25,26]. *MyHCIIb* may differ widely among breeds. Compared with that in crossbred pigs, the proportion of type I fibers was higher in native-breed pigs, which may have resulted from intensive selection [27]. We found that hybridization decreased the *MyHCI* expression in sheep muscle fibers. The high proportion of type IIb fibers tended to result in pale soft exudative pork [28], and hybridization significantly decreased the *MyHCIIb* expression in sheep muscle fibers, suggesting that hybridization can improve mutton quality.

The FAC of meat contributes greatly to its sensory properties, nutritional value, and all aspects of human health [29]. Health professionals worldwide recommend reducing SFA intake, and studies have shown a strong positive correlation between SFA intake and cardiovascular disease incidence [30]. Herein, the SFA content was significantly higher in the NH and SH groups than in the HH group, indicating that HH mutton was more beneficial to human health. The C18:0 content in SFAs may be the main reason for this difference. Although C18:0 does not affect serum cholesterol concentrations in the human body, C18:0 content is directly related to fat hardness [31,32]. Fat hardness increases as the C18:0 content increases, which further affects the fat melting point and juiciness of the meat [31]. Additionally, C12:0 increased the total cholesterol [30], which was significantly higher in the HH group than in the NH and SH groups. The MUFA content was significantly higher in the HH group than in the NH and SH groups, and the melting point of MUFAs is lower than that of SFAs, which helps in producing high-quality meat [33]. For MUFAs, the C18:1n9c content was significantly higher in the HH group than in the NH and SH groups. C18:1n9c lowers cholesterol and benefits human health [34]. PUFAs are beneficial fatty acids for humans. Higher PUFA contents help improve meat taste and lipid oxidation [35]. For PUFAs, the C18:2n6c and C18:3n3 contents were significantly higher in the NH and SH groups than in the HH group. C18:2n6c and C18:3n3 are essential fatty acids in the body; they must be obtained through food and have neuroprotective and anti-aging functions [36]. SFA and MUFA proportions increase and PUFAs decrease as body weight increases [37,38]. Compared with the HH group, the NH and SH groups had higher body weights, but their MUFA contents were inconsistent with previous findings. MUFA composition and contents in ruminants depend mainly on diet and may be caused by dietary compositions [29,39]. The SFA/PUFA and n-6PUFA/n-3PUFA ratios determine the nutritional value of meat. A PUFA/SFA ratio of ≥0.4 and an n-6/n-3 ratio of ≤4:1 have been recommended for meat [40]. Here, the PUFA/SFA ratio was <0.4, which is obviously lower than the recommended range, and the n-6/n-3 ratio was <4, which is higher than the recommended range. Previous studies have found that the PUFA/SFA and n-6/n-3 ratios in mutton are beyond the recommended range [41,42]; thus, mutton may be a poor source of dietary fatty acids.

Meat flavor is determined largely by VFCs, which are mainly generated from lipids and water-soluble compounds via the Maillard reaction and lipid oxidation upon processing [43]. Here, we identified 25 VFCs, with the largest proportion being aldehydes; this was the same as that of other VFCs in meat [44,45]. Because of their low odor detection threshold, aldehydes greatly affect the aroma of cooked meat [46]. Aldehydes can be formed by oxidation of UFAs or by degradation via Strecker amino acid synthesis [47]. Benzaldehyde was significantly differentially expressed in the three mutton hybrids; it has bitter almond flavor and can be formed by linolenic acid degradation, which occurs in cooked meat [48]. Hexanal is the oxidation product of C18:2n6, which is widely known in the food flavor industry for its clear, grassy flavor, but it produces an unpleasant, rotten smell when in high concentrations [49]. Heptanal is mainly produced by oxidation of linoleic acid and has been shown to be a key volatile compound in previous studies [41]. Ketones are mainly formed by oxidation of fatty acids, with obvious milk or fruit flavors, a relatively high odor threshold, and a lesser contribution to meat aromas [45]. We found that 2-butanone was differentially expressed in the three mutton hybrids and is usually considered to be a product of the Maillard reaction [50]. Alcohols are mainly produced by automatic fat oxidation in meat. Alcohols have a low odor threshold and promote formation of meat flavor [51]. We detected only two esters in this study, and their odor threshold was relatively high, but they contributed little to the meat flavor [45]. Pyrazines are final products of the Maillard reaction; they have a low odor threshold and contribute to producing baking flavor, but we detected only one pyrazine in this study [52].

## 5. Conclusions

We analyzed the effects of different hybrid sheep sires on MFCs, FAC, and VFCs in the F_1_ offspring. Compared with those of Hu sheep, the muscle fiber diameter, cross-sectional area, collagen fiber content, and fiber type were significantly altered in the hybrid lambs. Regarding FAC, the SFA contents were increased significantly in the hybrid lambs, whereas the MUFA contents were decreased significantly, and the FAC of the hybrid lambs was less favorable for human health. We identified eleven differentially expressed VFCs from the three mutton hybrids, and PCA enabled distinguishing among the hybrids. Our results provide insight into how hybridization affects lamb meat quality characteristics and may help guide production of high-quality mutton.

## Figures and Tables

**Table 1:** Composition and content of fatty acids in F_1_ crossbred lambs.

Types of Fatty Acids	HH Group	NH Group	SH Group	*p* Value
C10:0	8.50 ± 0.27 ^a^	5.20 ± 0.53 ^b^	5.98 ± 0.45 ^b^	<0.001
C12:0	7.55 ± 1.55 ^a^	4.40 ± 0.82 ^b^	3.23 ± 0.77 ^b^	0.008
C14:0	4.10 ± 0.69	3.29 ± 0.49	3.82 ± 0.06	0.197
C15:0	45.91 ± 1.59	42.81 ± 0.59	41.15 ± 0.16	0.081
C16:0	4.94 ± 0.62	3.82 ± 0.31	4.54 ± 0.48	0.074
C17:0	2.92 ± 0.57	2.37 ± 0.31	2.36 ± 0.06	0.201
C18:0	544.18 ± 51.56 ^c^	660.22 ± 22.91 ^b^	730.72 ± 14.36 ^a^	0.001
C20:0	3.45 ± 0.34 ^a^	1.80 ± 0.48 ^b^	1.63 ± 0.30 ^b^	0.002
C21:0	3.95 ± 0.21	3.48 ± 0.28	3.46 ± 0.27	0.094
C22:0	6.61 ± 0.57	6.61 ± 0.69	6.73 ± 0.72	0.970
SFA	632.13 ± 47.12 ^c^	733.99 ± 21.69 ^b^	803.62 ± 15.30 ^a^	0.002
C14:1	26.91 ± 1.78 ^a^	13.16 ± 0.90 ^b^	15.04 ± 0.42 ^b^	<0.001
C15:1	4.99 ± 0.87	3.90 ± 0.65	4.47 ± 0.44	0.085
C16:1	3.17 ± 0.20	2.94 ± 0.27	3.20 ± 0.46	0.581
C17:1	3.63 ± 0.16	3.40 ± 0.38	2.97 ± 0.23	0.074
C18:1n9c	13.63 ± 0.45 ^a^	6.20 ± 0.61 ^c^	8.07 ± 0.55 ^b^	<0.001
C18:1n9t	2314.24 ± 219.60 ^a^	1453.49 ± 93.39 ^b^	1846.77 ± 134.55 ^b^	0.002
C20:1	27.95 ± 0.67 ^a^	6.45 ± 1.29 ^b^	8.38 ± 1.02 ^b^	<0.001
C22:1n9	107.29 ± 6.55 ^a^	88.61 ± 4.42 ^b^	87.95 ± 3.35 ^b^	0.005
MUFA	2501.82 ± 219.52 ^a^	1578.15 ± 90.48 ^c^	1976.65 ± 136.40 ^b^	0.001
C18:2n6c	4.48 ± 0.14 ^b^	6.11 ± 0.33 ^a^	6.26 ± 0.03 ^a^	<0.001
C18:2n6t	10.98 ± 0.17 ^a^	7.91 ± 0.73 ^b^	8.72 ± 1.30 ^b^	0.012
C18:3n3	4.07 ± 0.37 ^c^	6.26 ± 0.55 ^b^	7.89 ± 0.60 ^a^	<0.001
C20:4n6	77.15 ± 2.44	77.38 ± 2.37	79.58 ± 2.86	0.483
C20:2	10.88 ± 0.38 ^a^	8.06 ± 0.73 ^b^	7.32 ± 0.42 ^b^	<0.001
PUFA	107.56 ± 2.00	105.72 ± 3.20	109.77 ± 2.24	0.226
PUFA/SFA	0.17 ± 0.02 ^a^	0.14 ± 0.01 ^b^	0.14 ± 0.01 ^b^	0.009
n-6/n-3	22.89 ± 2.55 ^a^	14.69 ± 1.44 ^b^	12.02 ± 0.75 ^b^	0.001

MUFAs: monounsaturated fatty acids; PUFAs: polyunsaturated fatty acids; SFAs: saturated fatty acids. In the same row, values with different lowercase mean significant difference (*p* < 0.05).

**Table 2:** Volatile flavor compounds tested in F_1_ crossbred lambs using GC–IMS.

Volatiles	Compounds	RI	Rt (s)	Dt (ms)	Intensity (V)			*p* Value
					HH	NH	SH	
Alcohols	1-Propanol	557.0	103.0	1.118	422.27 ± 89.71 ^b^	457.55 ± 108.65 ^b^	883.67 ± 194.27 ^a^	0.009
	1-Pentanol (M)	751.0	208.7	1.255	368.86 ± 106.43	586.77 ± 492.17	120.78 ± 26.68	0.226
	1-Pentanol (D)	749.9	207.8	1.520	58.21 ± 25.47	463.84 ± 652.28	62.45 ± 24.37	0.379
Aldehyde	Octanal	999.7	536.6	1.407	2401.29 ± 123.86	2478.25 ± 185.01	2351.89 ± 109.99	0.583
	Benzaldehyde (M)	948.3	447.6	1.150	987.45 ± 109.72 ^b^	1429.16 ± 169.29 ^a^	1325.33 ± 65.83 ^a^	0.002
	Benzaldehyde (D)	947.3	445.9	1.467	896.17 ± 163.05 ^b^	1255.74 ± 153.61 ^a^	1241.86 ± 177.03 ^a^	0.011
	Methional	897.1	360.2	1.091	992.68 ± 144.87 ^a^	573.80 ± 116.22 ^b^	488.91 ± 98.45 ^b^	0.026
	Heptanal	890.8	350.3	1.334	1902.59 ± 192.42 ^a^	1919.41 ± 177.39 ^a^	1663.95 ± 109.15 ^b^	0.027
	Hexanal (M)	784.0	234.9	1.562	1943.18 ± 193.30	1921.06 ± 178.00	1877.59 ± 168.18	0.621
	Hexanal (D)	784.0	234.9	1.265	1538.80 ± 172.17 ^a^	1483.36 ± 175.62 ^a^	884.48 ± 108.56 ^b^	0.008
	Pentanal (M)	688.8	159.3	1.191	2227.79 ± 182.03	2283.46 ± 210.81	1827.18 ± 229.91	0.083
	Pentanal (D)	688.0	158.9	1.422	1978.37 ± 153.23 ^a^	1299.80 ± 115.09 ^b^	118.66 ± 32.06 ^c^	<0.001
	3-Methylbutyraldehyde (M)	642.5	139.5	1.402	3173.46 ± 219.09 ^a^	2415.42 ± 109.40 ^b^	2354.49 ± 146.77 ^b^	0.016
	3-Methylbutyraldehyde (D)	638.8	137.9	1.180	1563.54 ± 138.59 ^a^	1168.37 ± 161.49 ^b^	852.65 ± 120.29 ^b^	0.024
	Butanal	612.1	126.5	1.286	196.79 ± 56.85	639.46 ± 576.82	159.08 ± 67.69	0.231
ketones	2-Pentanone (M)	676.8	154.1	1.368	2621.22 ± 188.28	2336.56 ± 190.78	2640.25 ± 167.31	0.261
	2-Pentanone (D)	676.8	154.1	1.120	2348.94 ± 187.85	2059.00 ± 198.72	2326.53 ± 178.79	0.113
	2-Butanone (M)	583.5	114.3	1.062	1594.18 ± 82.19 ^b^	1851.37 ± 115.61 ^a^	1875.26 ± 97.72 ^a^	0.033
	2-Butanone (D)	578.4	112.1	1.245	1361.33 ± 88.11 ^a^	1122.68 ± 32.88 ^b^	1153.82 ± 55.72 ^b^	0.012
	2,3-Butanedione	568.6	107.9	1.182	559.20 ± 58.12	486.18 ± 85.84	909.27 ± 312.03	0.071
	3-Hydroxy-2-butanone (M)	715.2	180.3	1.062	719.99 ± 242.96	276.36 ± 322.07	969.29 ± 318.31	0.392
	3-Hydroxy-2-butanone (D)	707.5	174.2	1.331	195.28 ± 193.46	173.39 ± 210.59	656.73 ± 536.55	0.242
Esters	Methyl 2-methylbutyrate	766.4	220.9	1.172	226.19 ± 174.65	77.52 ± 14.63	553.35 ± 656.28	0.376
	Methyl acetate	529.6	91.3	1.184	1515.07 ± 198.75	1613.22 ± 116.18	1461.68 ± 105.67	0.388
Pyrazines	2-Ethyl-5-methylpyrazine	1000.1	537.4	1.672	2824.14 ± 278.11	2963.25 ± 123.54	2949.90 ± 169.36	0.504

RI: retention index; Rt: retention times; Dt: drift times; M: monomer; D: dimer. In the same row, values with different lowercase mean significant difference (*p* < 0.05).

## Data Availability

Data are contained within the article.

## Supplementary Materials

The following supporting information can be downloaded at: https://www.mdpi.com/article/10.3390/foods11244076/s1, Table S1: Primers used in this study for qRT-PCR; Figure S1: Color deconvolution pictures of collagen fibers. Scale bars, 400 μm. HH: Hu × Hu, NH: Southdown × Hu, SH: Suffolk × Hu.
