# Supplementary material for: Effects of Sheep Sires on Muscle Fiber Characteristics, Fatty Acid Composition and Volatile Flavor Compounds in F1 Crossbred Lambs
Source: Foods. 2022 Dec 16;11(24):4076. doi: 10.3390/foods11244076 (PMC9778286; doi:10.3390/foods11244076)
Supplement: Supplementary file 1 [file foods-11-04076-s001.zip › foods-2061917-supplementary/Supplementary Files/Table S1.docx]

**Table S1.** Primers used in this study for qRT-PCR.

| Gene | Primer sequences (5'-3') | Products length (bp) |
| --- | --- | --- |
| *MyHCI* | AAGAACCTGCTGCGGCTG | 220 |
|  | CCAAGATGTGGCACGGCT |  |
| *MyHCIIa* | GAGGAACAATCCAATACAAATCTATCT | 192 |
|  | CCCATAGCATCAGGACACGA |  |
| *MyHCIIb* | GACAACTCCTCTCGCTTTGG | 218 |
|  | GGACTGTGATCTCCCCTTGA |  |
| *MyHCIIx* | GGAGGAACAATCCAATGTCAAC | 178 |
|  | GTCACTTTTTAGCATTTGGATGAGTTA |  |
| *ACTB* | CCAACCGTGAGAAGATGACC | 97 |
|  | CCCGAGGCGTACAGGGACAG |  |
