# Supplementary figures and images for: Effects of Sheep Sires on Muscle Fiber Characteristics, Fatty Acid Composition and Volatile Flavor Compounds in F1 Crossbred Lambs
Source: Foods. 2022 Dec 16;11(24):4076. doi: 10.3390/foods11244076 (PMC9778286; doi:10.3390/foods11244076)

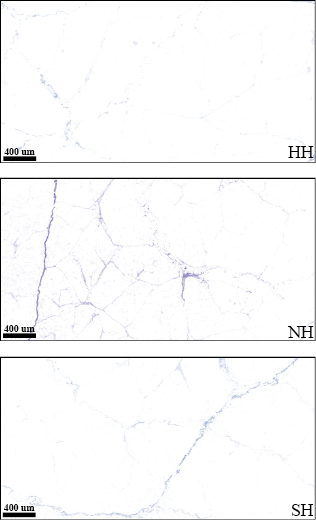

Supplement: Supplementary file 1 [file foods-11-04076-s001.zip › foods-2061917-supplementary/Supplementary Files/Figure S1.png]
